# A Retrospective Cohort Study of Traumatic Brain Injury in Children: A Single-Institution Experience and Determinants of Neurologic Outcome

**DOI:** 10.2478/jccm-2023-0027

**Published:** 2023-11-14

**Authors:** Merve Misirlioglu, Faruk Ekinci, Dincer Yildizdas, Ozden Ozgur Horoz, Hayri Levent Yilmaz, Faruk Incecik, Mazhar Ozsoy, Ahmet Yontem, Sevcan Bilen, Sena Silay

**Affiliations:** Department of Pediatric Intensive Care, Mersin University Faculty of Medicine, Mersin, Turkey; Department of Pediatric Intensive Care, Cukurova University Faculty of Medicine, Adana, Turkey; Department of Pediatric Emergency, Cukurova University Faculty of Medicine, Adana, Turkey; Department of Pediatric Neurology, Cukurova University Faculty of Medicine, Adana, Turkey; Department of Neurosurgery, Cukurova University Faculty of Medicine, Adana, Turkey; Department of Pediatrics, Cukurova University Faculty of Medicine, Adana, Turkey

**Keywords:** pediatric trauma, traumatic brain injury, children, enteral nutrition, outcomes

## Abstract

**Introduction:**

Traumatic brain injury (TBI) has become a significant cause of death and morbidity in childhood since the elucidation of infectious causes within the last century. Mortality rates in this population decreased over time due to developments in technology and effective treatment modalities.

**Aim of the study:**

This retrospective cohort study aimed to describe the volume, severity and mechanism of all hospital-admitted pediatric TBI patients at a university hospital over a 5-year period.

**Material and Methods:**

This was a single-center, retrospective cohort study including 90 pediatric patients with TBI admitted to a tertiary care PICU. The patients’ demographic data, injury mechanisms, disease and trauma severity scores, initiation of enteral nutrition and outcome measures such as hospital stay, PICU stay, duration of mechanical ventilation, mortality, and Glasgow Outcome Scale (GOS) were also recorded. Late enteral nutrition was defined as initiation of enteral feeding after 48 hours of hospitalization.

**Results:**

Of the 90 patients included in the cohort, 60% had mild TBI, 21.1% had moderate TBI and 18.9% had severe TBI. Their mean age was 69 months (3–210 months). TBI was isolated in 34 (37.8%) patients and observed as a part of multisystemic trauma in 56 (62.2%). The most commonly involved site in multisystemic injury was the thorax (33.3%). The length of hospitalization in the late enteral nutrition group was significantly higher than that in the early nutrition group, while the PICU stay was not significantly different between the two groups. The multiple logistic regression analysis found a significant relationship between GOS-3rd month and PIM3 score, the presence of diffuse axonal injury and the need for CPR in the first 24 h of hospitalization.

**Conclusion:**

Although our study showed that delayed enteral nutrition did not affect neurologic outcome, it may lead to prolonged hospitalization and increased hospital costs. High PIM3 scores and diffuse axonal injury are both associated with worse neurologic outcomes.

## Introduction

Traumatic brain injury (TBI) has become a significant cause of death and morbidity in childhood since the elucidation of infectious causes within the last century [1]. Despite efforts to reduce its incidence, TBI remains a major problem in pediatrics. TBI accounts for 32.7% of all trauma cases and TBI mortality rates vary between 0.4 and 4.2% in different studies [1,2,3,4]. In addition to this variability, causes of pediatric TBI vary between different countries, even in distinct geographical regions of the same country. The most frequent causes of TBI are falls and traffic accidents, of which the latter is more common in adolescence. Severe TBI necessitates intensive care unit hospitalization and a multidisciplinary approach, as patients usually have additional system injuries [5]. TBI management is usually supportive and includes optimal ventilation, maintenance of fluid-electrolyte homeostasis, optimization of cerebral metabolism, as well as prevention of cerebral herniation and irreversible brain injury. Repetitive neurological assessments, appropriate brain imaging and surgical interventions in selected cases are the principal steps in TBI management [1,6].

A recent comprehensive study has shown that after a consistent decline, pediatric TBI mortality in the United States increased significantly after 2013 and was linked to suicide mortality [7]. In general, mortality rates in this population decreased over time due to developments in technology and effective treatment modalities. However, morbidity, particularly neurologic sequelae, is one of the most devastating problems affecting these patients. The Glasgow Outcome Scale (GOS) allows for an overall outcome assessment after TBI based on objective criteria [8]. The factors affecting neurologic outcomes in pediatric TBI are still unclear and need to be further evaluated.

Nutrition guidelines recommend that critically ill children hospitalized in intensive care should be evaluated and enteral nutrition should be started if there is no contraindication within the first 24–48 hours. Feeding started after 48 hours is considered as late enteral feeding [9]. Early enteral feeding is common in critically ill children after TBI. Children with severe TBI are more likely to have delayed initiation of enteral nutrition. Abdominal injury and procedures are associated with delayed enteral nutrition in unadjusted analysis, though not after adjustment for injury/illness severity. Delayed enteral nutrition is also an independent risk factor for worse functional status at PICU discharge [10]. Therefore, we aimed to conduct a renewed study investigating the causes and outcomes of TBIs admitted to our pediatric intensive care unit (PICU) within the last five years. We also evaluated factors associated with late enteral nutrition and worse neurologic outcomes at 3^rd^ month after trauma.

## Materials and Methods

### Patient selection

This retrospective cohort study included children with TBI hospitalized in our PICU between January 2015 and December 2019. Their demographic features, including age and sex, weight, trauma etiology, pediatric risk of mortality score (PRISM), pediatric index of mortality 3 (PIM3), injury severity score (ISS), pediatric trauma score (PTS), Glasgow Coma Scale (GCS), pupil reflexes, initiation time of enteral feeding, computerized tomography and electroencephalogram results were recorded retrospectively from the medical files of the patients [11,12,13]. Data on the treatment procedures, including mechanical ventilation support and antiedema treatments such as hypertonic solutions, anticonvulsant agents and blood transfusions were also recorded. Neurological injury severity was assessed by GCS and the patients were grouped by severe (GCS: 3–8), moderate (GCS: 9–12) and mild (GCS: 13–15) injury according to GCS at emergency care department presentation.

The initiation of enteral feeding was also investigated. Enteral feeding was categorized as early if it started before the 48^th^ hour of hospitalization and late if it started after this time. The outcomes of TBI patients were evaluated according to GOS-discharge and GOS-3^rd^ month. GOS at three months was classified as a favorable outcome if it was equal to five or disability if below five. The impacts of demographic and clinical features, PRISM-III score, PIM3, ISS, PTS, and GCS on enteral feeding initiation and outcome were investigated. Enteral feeding was also tested as a determinant for the patients’ outcomes.

The study was ethically approved by the Cukurova University Institutional Research Ethics Committee (Date: March 6, 2020, Number: 97/27). The need for informed consent for participation was waived, as this was a retrospective confidential medical record review.

### Statistical Analysis

We performed all statistical analyses using the SPSS 20.0 statistical software (IBM SPSS Statistics). The distribution of variables was investigated using visual (histogram and probability plots) and analytical methods (Kolmogorov Smirnov test) to determine whether they are normally distributed or not. Descriptive analyses of continuous variables were presented using mean±standard deviations (SD) for normally distributed variables, whereas median and minimum-maximum values were used for non-normally distributed and ordinal variables. Comparative analyses between two groups were performed with the Mann Whitney U and Chi-Square tests. Categorical variables were expressed as numbers and percentages and were compared by performing a chi-square test between two groups. A p value < 0.05 was considered statistically significant. Multivariate logistic regression analysis with stepwise selection was used to identify independent predictors associated with worse GOS at 3^rd^ month after traumatic brain injury. The parameters (in Table 4) with a p value of smaller than 0.25 were included for further multivariable analysis in table 5. A cutoff value of 0.25 is supported by recent literature [14, 15].

## Results

**Fig. 1.**
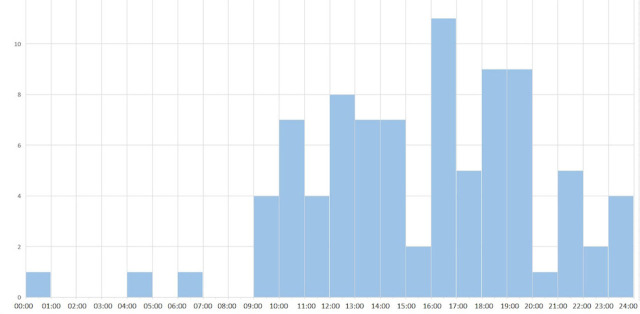
Distribution of trauma cases by hours of the day in TBI patients

**Fig. 2.**
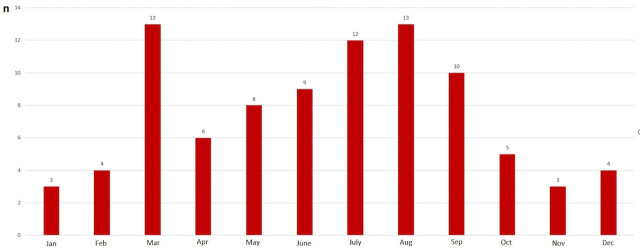
Distribution of trauma cases by months of the year in TBI patients

**Fig. 3.**
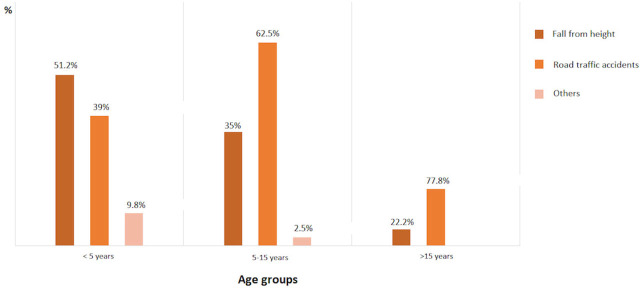
Etiology of TBI in different age groups

The study included 90 children, 37 (41.1%) females and 53 (58.9%) males. The mean age was 69 months (range: 3–210 months). Our center was the first place of admission for 56 (62.2%) patients and the other 34 (37.8%) patients were referred from another hospital. Of 90 patients, 66 (73.3%) were transported to our center by emergency ambulance services and 24 (26.7%) were admitted to the hospital using their own vehicles. The majority of TBI occurred during the daytime, between 9 a.m. and 8 p.m. (Figure 1). The majority of TBIs occurred in warm weather between June and September (Figure 2). The median admission time to the emergency unit and waiting time before transfer to the ICU were 75 minutes (range: 15 minutes–24 hours) and six hours (range: 10 minutes–29 hours), respectively. The median duration of ICU stay was three days (range: 1–47 days) and the median duration of total hospitalization was seven days (range: 2–60 days). Table 1 provides information about the causes and locations of trauma. The causes of trauma varied between ages ≤5, 5–15, and ≥15 years (Figure 3).

**Table 1.** The causes, locations, and clinical features of traumatic brain injury in 90 children

**Causes**	**n**	**%**	**Locations**	**n**	**%**
Road traffic accidents	39	43.3	Outdoor	67	74.4
Passenger	12	13.3	Home	21	23.3
Pedestrian	27	30	School	2	2.2
Fall from height	34	37.8	CT/MRI findings	n	%
Cyclist	9	10	Skull fracture	58	64.4
Falling object	4	4.4	Brain edema	44	48.9
Basic fall	3	3.3	Subdural hemorrhage	19	21.1
Other	1	1.1	Epidural hemorrhage	14	15.6
Sign and symptoms	n	%	Intraparenchymal hemorrhage	13	14.4
Altered consciousness	59	65.6	Brain contusion	10	11.1
Nausea/Vomiting	28	31.1	Subarachnoid hemorrhage	9	10
Seizure	16	17.8	Diffuse axonal injury	5	5.6
Headache	9	10	Minimal brain edema	5	5.6
Amnesia	2	2.2			
Asymptomatic	6	6.7			

CT; computerized tomography, MRI; magnetic resonance imaging, TBI; traumatic brain injury

According to GCS at hospitalization, 54 (60%) had mild (GCS: 13–15), 19 (21.1%) had moderate (GCS: 9–12) and 17 (18.9%) had severe (GCS: ≤8) TBI. TBI was isolated in 34 (37.8%) cases and accompanied by injuries to other body parts in 56 (62.2%) patients. The most common area involved in multisystem injury was the thorax (n=30, 33.3%), which was followed by extremities (n=26, 28.9%), face (n=23, 25.6%), abdomen (n=21, 23.3%), pelvis (n=8, 8.9%), and vertebra (n=8, 8.9%).

The most common symptom was altered consciousness, which was present in 59 (65.6%) patients. Although 6 (6.7%) of the patients did not report any neurological symptoms, they routinely underwent brain imaging due to severe multisystem injuries and were diagnosed with TBI according to their radiological findings. The most common neuroradiological findings were skull fracture and brain edema, present in 58 (64.4%) and 44 (48.9) patients, respectively. Skull fractures were located on the calvaria in 42 (46.7%), face in 13 (14.4%) and skull base in 3 (3.3%) patients. Calvarium fractures were linear in 31 (71.8%) and depressed in 11 (26.2%) of 42 patients. Nonetheless, 5 of 90 (5.6%) patients did not have radiological evidence of TBI, despite their TBI clinical symptoms. Table 1 shows the clinical characteristics of the patients.

Electroencephalogram (EEG) was performed in 24 patients, and of these, 18 (75%) had an EEG abnormality. Five patients had an epileptiform pattern, and 13 patients had background rhythm abnormalities. Respiratory support was performed in 46 (51.1%) patients, and the majority were supported through a nonrebreathing oxygen mask (31.1%), while 16 patients were intubated and invasive mechanical ventilation was initiated. The median duration of mechanical ventilation was five days (range: 1–28 days). Treatment approaches are given in Supplemental Table 1. Enteral feeding was started during the first day in 12 (13.3%), the second day in 53 (58.9%), and the third day in 17 (18.9%) patients during their hospitalization. Enteral feeding was started after the third day in 8 (8.9%) patients. The median time of enteral nutrition initiation in the full cohort was two days (range: 1–7 days).

Nosocomial infections were encountered in 14 (15.5%) of the patients. The infections observed in the study cohort were as follows: bloodstream infections in six patients, nosocomial pneumonia in three patients, catheter-related bloodstream infection in two patients, nosocomial urinary tract infection in two patients and central nervous system infection in one patient. Only one patient died on the 28th day of hospitalization, with a 30-day mortality rate of 1.1% in the full cohort. Nine (10%) patients were rehospitalized, with one (1.1%) patient readmitted to the PICU at the one-year follow-up.

The patients were grouped according to the presence of early enteral nutrition, defined as the initiation of enteral nutrition during the first 48 hours of hospitalization. Multisystemic trauma, including extremity, pelvis, abdomen and facial trauma, was statistically higher in the late enteral nutrition group (p<0.05). There was no statistically significant difference between the early and late nutrition groups in terms of CT and MRI findings of the central nervous system. Blood product transfusion and noncranial surgery requirements in the first 24 hours after trauma were higher in the late enteral nutrition group (respectively, p=0.043, 0.001). The length of hospitalization in the late enteral nutrition group was significantly higher than that in the early nutrition group (p=0.001). However, the PICU stay was not significantly different between the two groups (Table 2). Furthermore, the median ISS was significantly higher in patients who started enteral nutrition after the 48th hour of hospitalization (p=0.01). The median PRISM, PDR, PIM3, PTS, GCS, GOS-discharge and GOS-3rd month did not differ between these two groups (Table 3).

**Table 2.** Comparison of demographic and clinical parameters between patients according to the presence of early, and late enteral feeding

	**<48^th^ hour of hospitalization (n=65)**	**>48^th^ hour of hospitalization (n=25)**	**p**
Age (year), median	5.08 (0.25–17.5)	7.58 (0.67–17.5)	0.063
Gender (female)	25 (38.5)	12 (48)	0.410

Trauma localization			
Multisystemic trauma	32 (49.2)	24 (96)	0.001
Extremity trauma	11 (16.9)	15 (60)	0.001
Pelvic trauma	1 (1.5)	7 (28)	0.001
Thorax trauma	20 (30.8)	10 (40)	0.405
Abdominal trauma	8 (12.3)	13 (52)	0.001
Facial trauma	11 (16.9)	12 (48)	0.002
Vertebral trauma	4 (6.2)	4 (16)	0.142

CT/MRI findings			
Skull fracture	40 (61.5)	18 (72)	0.353
Brain contusion	7 (10.8)	3 (12)	0.868
Subarachnoid hemorrhage	5 (7.7)	4 (16)	0.239
Subdural hemorrhage	15 (23.1)	4 (16)	0.461
Epidural hemorrhage	11(16.9)	3 (12)	0.564
Intraparenchymal hemorrhage	10 (15.4)	3 (12)	0.682
Brain edema	31 (47.7)	13 (52)	0.714
Diffuse axonal injury	5 (7.7)	0	0.067

First 24 hours			
Blood products	19 (29.2)	13 (52)	0.043
Cranial surgery	8 (12.3)	2 (8)	0.560
Other surgery	6 (9.2)	11 (44)	0.001
CPR	1 (1.5)	2 (8)	0.128
Mechanical ventilation	11 (16.9)	5(20)	0.732
Nosocomial infection	10 (15.4)	4 (16)	0.943
PICU stay	3 (1–28)	5 (1–47)	0.272
Length of hospitalization	5 (2–43)	10 (5–60)	0.001

**Table 3.** Comparison of different scoring systems of the TBI patients according to the presence of early, and late enteral feeding

**Parameter**	**Enteral feeding**		**p**
	**<48^th^ hour of hospitalization (n=65)**	**>48^th^ hour of hospitalization (n=25)**	
PRISM	5 (0–25)	6 (1–27)	0.124
PDR (%)	1.6 (0.5–59)	2.1 (0.4–52.8)	0.296
PIM3	2 (0.7–27)	2.2 (0.9–11)	0.182
Pediatric Trauma Score	10 (−1–12)	9 (1–12)	0.139
Injury severity score	5 (1–50)	10 (2–41)	0.001
GCS	13 (2–15)	13 (5–15)	0.985
GOS at discharge	5 (1–5)	5 (3–5)	0.340
GOS at 3^rd^ month	5 (1–5)	5 (3–5)	0.238
Favorable GOS at discharge	46 (70.8)	15 (60)	0.327
Favorable GOS at 3^rd^ month	50 (76.9)	16 (64)	0.214

PRISM; The Pediatric Risk of Mortality score, PDR; predicted death rate, PIM3; The Pediatric Index of Mortality 3, GCS; Glasgow coma scale, GOS: Glasgow outcome score

Among demographic and clinical parameters, multisystemic trauma, thorax trauma, altered consciousness in the emergency department (ED), seizures, brain contusion, subarachnoid hemorrhage, subdural hemorrhage, diffuse axonal injury, inotropic drugs in the ED, blood products in the first 48 hours, cranial surgery in the first 24 hours, CPR and MV in the first 24 hours of hospitalization, and nosocomial infection were statistically more frequent in patients with a GOS-3rd month below five (p<0.05). Similarly, the median stay at PICU, length of hospitalization, PRISM, PDR, PIM3, and ISS were statistically higher, and in contrast, the median trauma score and GCS were statistically lower in the worse outcome group (p=0.001) (Table 4). The multiple logistic regression analysis showed a significant relationship between the outcome and PIM3 score, the presence of diffuse axonal injury, and the need for CPR in the first 24 hours of hospitalization (p<0.05) (Table 5).

**Table 4.** Comparison of demographic and clinical properties between patients with favorable outcome and disability according to Glasgow Outcome Score (GOS) 3 months after TBI

**Parameter**	**GOS at 3^rd^ month**		**p**
	**GOS=5 (n=66)**	**GOS≤4 (n=24)**	
Age	4.9 (0.3–17.5)	6.1 (0.2–17.5)	0.942
Gender (female)	29 (43.9)	8 (33.3)	0.366
Multisystemic trauma	37 (56.1)	19 (79.2)	**0.046**

Trauma localization in multisystemic trauma			
Extremity trauma	20 (30.3)	6 (25)	0.624
Pelvic trauma	6 (9.1)	2 (8.3)	0.911
Thorax trauma	14 (21.2)	16 (66.7)	**0.001**
Abdominal trauma	12 (18.2)	9 (37.5)	0.054
Facial trauma	17 (25.8)	6 (25)	0.942
Vertebral trauma	7 (10.6)	1 (4.2)	0.342
Altered consciousness	37 (56.1)	22 (91.7)	**0.002**
Seizure	5 (7.6)	11 (45.8)	**0.001**

CT/MRI findings			
Skull fracture	46 (69.7)	12 (50)	0.084
Brain contusion	26 (39.4)	23 (95.8)	**0.001**
Subarachnoid hemorrhage	4 (6.1)	5 (20.8)	**0.039**
Subdural hemorrhage	9 (13.6)	10 (41.7)	**0.004**
Epidural hemorrhage	9 (13.6)	5 (20.8)	0.405
Intraparenchymal hemorrhage	7 (10.6)	6 (25)	0.086
Brain edema	31 (47)	13 (54.2)	0.546
Diffuse axonal injury	0	5 (28)	**0.001**
Shock treatment in ED	5 (7.6)	5 (20.8)	0.077
Inotropic drug in ED	1 (1.5)	3 (12.5)	**0.025**
Blood products in 48 h	16 (24.2)	16 (66.7)	**0.001**
Cranial surgery in 24 h	4 (6.1)	6 (25)	**0.011**
Other surgery in 24 h	11 (16.7)	6 (25)	0.372
CPR in the first 24h of hospitalization	0	3 (12.5)	**0.003**
MV in the first 24h of hospitalization	1 (1.5)	15 (62.5)	**0.001**
Nosocomial infections	2 (3)	12 (50)	**0.001**
PICU stay	2 (1–12)	9 (2–47)	**0.001**
Length of hospitalization	5 (2–33)	16.5 (6–60)	**0.001**
MV duration	1 (1–4)	5 (1–28)	0.061
PRISM	4 (0–18)	16 (4–27)	**0.001**
PDR (%)	1.5 (0.4–25.5)	15.5 (0–59)	**0.001**
PIM3	1.8 (0.7–8)	6.8 (1.2–27)	**0.001**
Pediatric Trauma score (PTS)	10 (6–12)	5 (−1–11)	**0.001**
Injury severity score (ISS)	5 (1–24)	15 (4–50)	**0.001**
GCS at admission	15 (2–15)	8 (4–15)	**0.001**

**Table 5.** Multiple logistic regression analysis of variables associated with outcome according to the 3rd month GOS in traumatic brain injury patients

**Variables**	**B**	**S.E.**	**P**	**OR (95% CI)**
Diffuse axonal injury	−0.281	0.243	**0.001**	2.62 (1.83–3.74)
CPR in the first 24h of hospitalization	−0.339	0.503	**0.005**	1.62 (1.13–2.74)
PIM3	−0.285	0.023	**0.023**	1.56 (0.40–6.19)

GOS; Glasgow Outcome Score, CPR; cardiopulmonary resusitaition, PIM3; The Pediatric Index of Mortality 3.

## Discussion

We reported a retrospective cohort of 90 pediatric TBI patients hospitalized in our PICU over a five-year period. Overall, the majority of TBIs occurred outdoors and road traffic accidents accounted for the most common cause, followed by falls from a height. Road traffic accidents were more frequently encountered with increasing age, while falls from height diminished. The most common symptom was altered consciousness, while the most frequent neuroradiological abnormalities were skull fracture and brain edema. Early enteral nutrition during the first 48 hours of hospitalization was administered to 72.2% of the patients. Multisystemic trauma (including extremity, pelvis, abdomen, and face), blood product requirement, and noncranial surgery were observed more frequently in the late enteral nutrition group. The length of hospitalization and ISS were significantly higher in this group. In addition, we found a significant relationship between lower GOS scores at the third month and the presence of diffuse axonal injury, CPR in the first 24 hours of hospitalization and higher PIM3 scores.

Although TBI usually occurs as minor injuries in childhood, it can result in significant mortality and morbidity, including long-term disability and intellectual, personality and behavioral problems. Falls are the most common cause of TBI during childhood worldwide, although the type of fall differs between geographical regions [16,17,18,19]. In our study, road traffic accidents (43.3%) were the most common cause, whereas falls from height were encountered in 37.8% of patients. We believe that this can be attributed to road traffic accidents requiring more PICU hospitalization.

Various studies worldwide have revealed a male predominance in TBI with a range of 59.2–78% (1–4,16–19). Not striking, but similarly, 58.9% of our cohort was male. Traffic accidents were shown to cause TBI in 10.2–37.9% of the overall population [1,4,17,18]. Interestingly, Efendioglu et al. and Hawley et al. suggested that while the causes of TBI did not differ with gender, age was the primary determinant, with the proportion of traffic accidents increasing as age increased [4,18]. Similarly, the proportion of road traffic accidents increased with age in our study.

Kim et al. did not find seasonal variation between the causes of TBI in children [2]. However, Hawley et al. showed the lowest incidence of TBI in December and January and the highest incidence during school holidays [4]. Kim et al. suggested that the majority of injuries occurred during the afternoon and evening [2]. Similarly, we found that the frequency of TBI was lowest between November and February and it was most common during late afternoons, 4–5 p.m.

Mortality rates vary among different studies worldwide because mortality is strictly related to the severity of injury and comorbidities, which may also differ among studies. For example, decreased levels of consciousness accompanied by neck pain should alert the physician to a possible concomitant cervical spine injury and the spine should be immobilized. In addition, a GCS of less than nine and a fluctuating mental status should lead the physician to secure the airway [20,21]. In our study, 18.9% of the cohort had severely altered consciousness based on GCS, 17.8% of the patients required MV and the overall mortality rate was 1.1%. The mortality rates vary in different studies. An epidemiologic study from Norway on pediatric traumatic brain injuries reported a mortality rate of 1.7% in 176 children [22]. Another multicenter study reported a mortality rate of 2.6% in a sizeable pediatric study group consisting of 416 patients [12]. Our mortality rates were consistent with those reported in other studies from various parts of the world.

ICP monitoring is now recommended for the management of severe pediatric TBI. Previous reports have suggested a strong correlation between high ICP and morbidity, and recent studies have suggested that optimal cerebral perfusion pressure is linked to better outcomes in TBI [21,23,24]. Unfortunately, we could perform ICP monitoring in only one patient. We could not perform it routinely in cases of severe TBI due to technical difficulties and a lack of necessary materials in our center.

The therapeutic use of sedatives, analgesics and neuromuscular blockade to decrease ICP is also recommended during PICU hospitalization for severe cases. Intravenous mannitol and hypertonic saline should be used routinely to control intracranial hypertension (ICH) in severe pediatric TBI, and if unresponsive to these drugs, barbiturates are also recommended [21]. Furthermore, decompressive craniectomy (DC) can be performed in irresistible ICH, resulting in reduced mortality, but the long-term effects have not yet been clarified [25]. In our study, hypertonic saline was the most common therapeutic approach and decompressive surgery was performed in 11.1% of cases.

Delayed enteral feeding, defined as the initiation of enteral nutrition 48 hours after PICU admission, has recently been suggested to cause worse functional outcomes in pediatric TBI patients at discharge. Balakrishnan et al. reported early enteral feeding in 83% of their cohort. Low GCS and high ISS were more likely to have delayed initiation of enteral nutrition in the same study [12]. Early enteral feeding could be achieved in 72.2% of our pediatric TBI cohort. The lower rate of early enteral feeding may be due to a higher frequency of abdominal injury in our study (23.3%) compared to that study (5.5%) [12]. Only multisystemic injury, higher ISS, blood product transfusion and noncranial surgery requirement were associated with delayed enteral feeding. However, we were unable to show any relationship between early enteral nutrition and better neurologic outcomes in our study. Predicting the outcome of TBI has been challenging, and therefore, remains an area of investigation. Several predictors, including age, GCS, PRISM, pupillary reactivity, hypernatremia and CT characteristics (including the presence of cerebral edema, traumatic subarachnoid hemorrhage and multifocal contusions), have been proposed thus far [26,27,28]. In our study, we found that the PIM3 score, the presence of diffuse axonal injury, and CPR in the first 24 hours of hospitalization were independent predictive factors for the outcome of pediatric TBI patients. In addition, early enteral nutrition did not affect neurological outcomes.

Our study has some limitations, including the retrospective study design and small sample size. In addition, we do not have enough data about the long-term neurological outcomes of these patients. Nonetheless, we believe that being a comprehensive descriptive study and investigating both enteral nutrition and neurological outcome, of which predictors need to be clarified, makes the present study noteworthy. Further comprehensive, long-term, and prospective studies are needed to validate our results.

## Conclusions

Delayed enteral nutrition may be caused by multisystemic trauma and noncranial surgery. Although this does not affect the neurological outcome, it may lead to prolonged hospitalization and hospital costs. In addition, the presence of diffuse axonal injury, CPR in the first 24 hours of hospitalization and higher PIM3 scores can lead to a worse neurological outcome in pediatric TBI patients.
